# Anterior longitudinal ligament in diffuse idiopathic skeletal hyperostosis: Ossified or displaced?

**DOI:** 10.1002/jor.24020

**Published:** 2018-05-24

**Authors:** Jonneke S. Kuperus, Esther J. M. Smit, Behdad Pouran, Robbert W. van Hamersvelt, Marijn van Stralen, Peter R. Seevinck, Constantinus F. Buckens, Ronald L. A. W. Bleys, Harrie H. Weinans, F. Cumhur Oner, Pim A. de Jong, Jorrit‐Jan Verlaan

**Affiliations:** ^1^ Department of Orthopedic Surgery University Medical Center Utrecht Utrecht University Box 85500, 3508 GA Utrecht The Netherlands; ^2^ Department of Biomechanical Engineering Faculty of Mechanical, Maritime, and Materials Engineering, Delft University of Technology Delft The Netherlands; ^3^ Department of Radiology University Medical Center Utrecht Utrecht University Utrecht The Netherlands; ^4^ Image Science Institute Utrecht University Utrecht The Netherlands; ^5^ Department of Anatomy University Medical Center Utrecht Utrecht University Utrecht The Netherlands

**Keywords:** diffuse idiopathic skeletal hyperostosis, anterior longitudinal ligament, computed tomography, cryomacrotome, pathogenesis, spine, bone/bone biology

## Abstract

Diffuse idiopathic skeletal hyperostosis (DISH) is often theorized to be an ossification of the anterior longitudinal ligament (ALL). Using computed tomography (CT) imaging and cryomacrotome sectioning, we investigated the spatial relationship between the ALL and newly formed bone in DISH to test this hypothesis. In the current study, four human cadaveric spines diagnosed with DISH using CT imaging were frozen and sectioned using a cryomacrotome. Photographs were obtained of the specimen at 125 µm intervals. Manual segmentations of the ALL on cryomacrotome photographs were projected onto the three‐dimensional reconstructed CT scans. The presence and location of newly formed bone were assessed in relationship to the location of the ALL. The ALL could be identified and segmented on the photographs at all levels. The ALL was located at the midline at levels where no new bone had formed. At the locations where new bone had abundantly formed, the ALL was displaced towards to the contralateral side and not replaced by bony tissue. The displacement of the—morphologically normal appearing—ALL away from the newly formed bone implies that newly formed bone in DISH may not originate from the ALL. © 2018 The Authors. *Journal of Orthopaedic Research*® Published by Wiley Periodicals, Inc. on behalf of Orthopaedic Research Society J Orthop Res 36:2491–2496, 2018.

Diffuse idiopathic skeletal hyperostosis (DISH) is a condition defined as bridging ossifications over at least four contiguous vertebral bodies at the anterolateral spine according to the criteria of Resnick and Niwayama (Fig. 1).^1^, ^2^ The prevalence of DISH has been described to range between 2.9% and 42.0% depending on the classification criteria used and presence of risk factors in the studied population.^1^, ^3^ DISH is associated with older age, male gender, obesity, diabetes mellitus, and hypertension.^4^ Even though the pathogenesis of DISH has yet to be elucidated, mechanical, vascular, metabolic, and genetic factors as well as signaling pathways have been suggested as possible mechanisms influencing the growth of new bone in DISH.^5^

**Figure 1:**
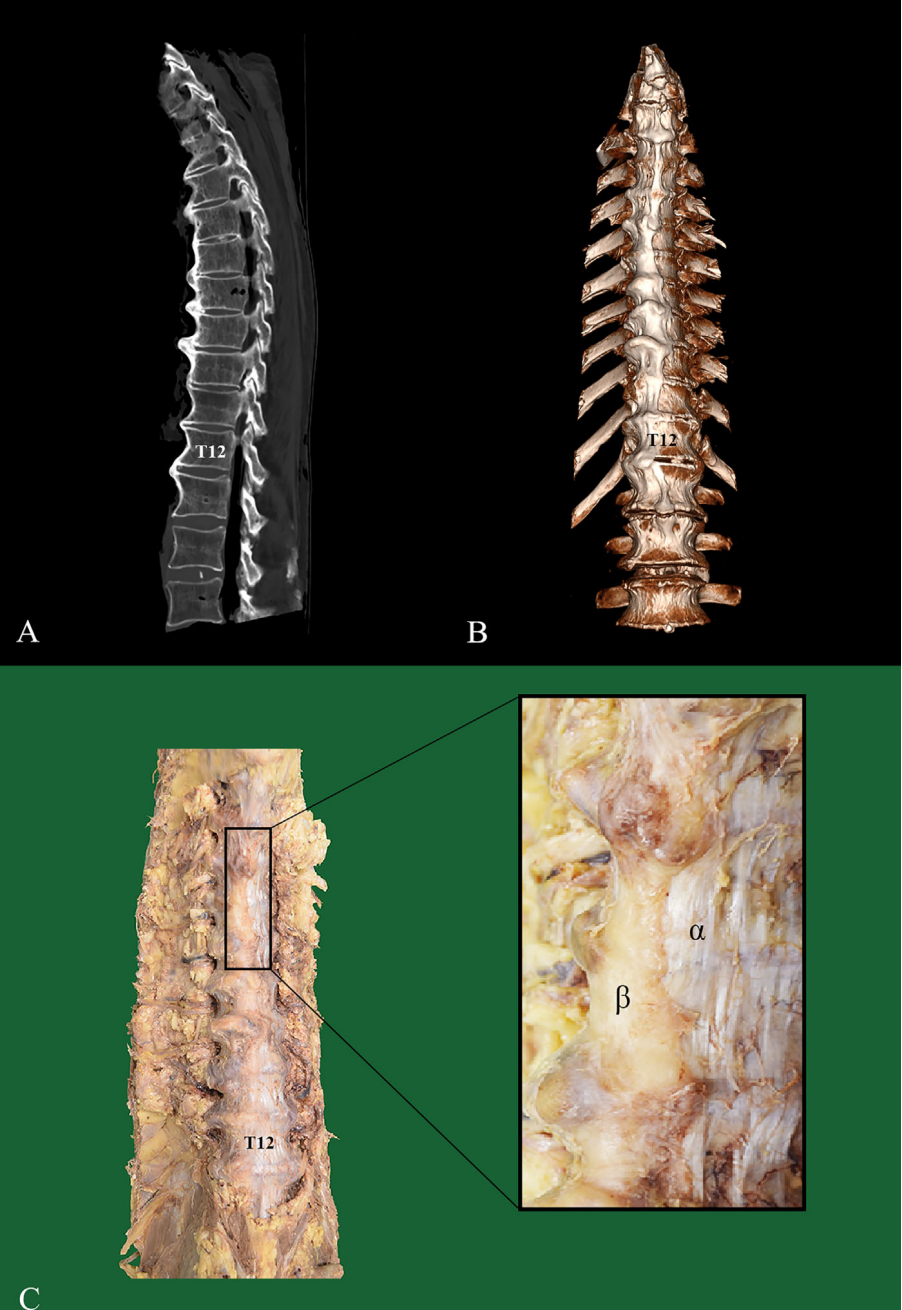
Example of a sagittal and three‐dimensional reconstructed CT scan and a matching macroscopic photograph of a spine with unequivocal DISH. In (A and B), the sagittal and three dimensional reconstructed CT scan of a male (88‐year‐old at time of death) is shown with newly formed bone consistent with the diagnosis DISH present on the right side of the thoracic spine. In (C), a photograph of the same specimen is shown, with special attention to the clear separation between the bone (β) and ALL (α) (levels T5‐T8). CT, computed tomography; ALL, anterior longitudinal ligament.

The anterior longitudinal ligament (ALL) is a relatively narrow continuous tendinous band located predominantly in the midsagittal frontal plane of the spinal column. Several authors have suggested the newly formed bone in DISH to be equivalent to, or originate from, ossification/calcification of the ALL.^5^, ^6^, ^7^, ^8^, ^9^ However, the predilection of the new bone to grow laterally of the midline suggests that the ALL may not be the (exclusive) origin of newly formed bone in DISH, or may not ossify at all.

The aim of this study was to investigate the hypothesis that DISH starts as an aberrant/degenerative ossification of the ALL by examining the spatial relationship between the ALL and the newly formed bone in human cadaveric spines with DISH using a combination of computed tomography (CT) imaging and cryomacrotome sectioning.

## MATERIALS AND METHODS

For this study, human torsos were used from persons who had entered the department of anatomy through a donation program. Written informed consent was obtained during life allowing the use of their entire bodies for educational and research purposes. Screening for DISH was performed by two observers using fluoroscopy examination (Omnidiagnost Eleva; Philips Medical Systems, Best, The Netherlands). Four thoracic spines with unequivocal DISH according to the Resnick criteria (without other apparent disease) and one spine without DISH (serving as control) were resected in toto with inclusion of at least T4 to L1 and approximately 5 cm of each rib.^2^ Surrounding soft tissues were removed taking care not to disturb the ALL. Computed tomography scans were acquired using a 64‐detector row spectral CT scanner (Appendix S1). The spines with DISH were photographed and macroscopically inspected for the presence of new bone and for identification of the ALL (Fig. 1). Subsequently, the DISH spines were cut into a cranial, middle and caudal section and were axially sectioned using a cryomacrotome with 25 µm slice thickness (Appendix S1). Photographs were taken (at 125 µm intervals) of the frozen blocks containing the specimens and slice sections were collected (at 500 µm intervals) on adhesive tape. For the cadaveric spine without DISH, three 20 × 10 mm rectangular fragments of the ALL were resected, processed with equivalent settings and included as a control.

### Sample Analyses

**Figure 2:**
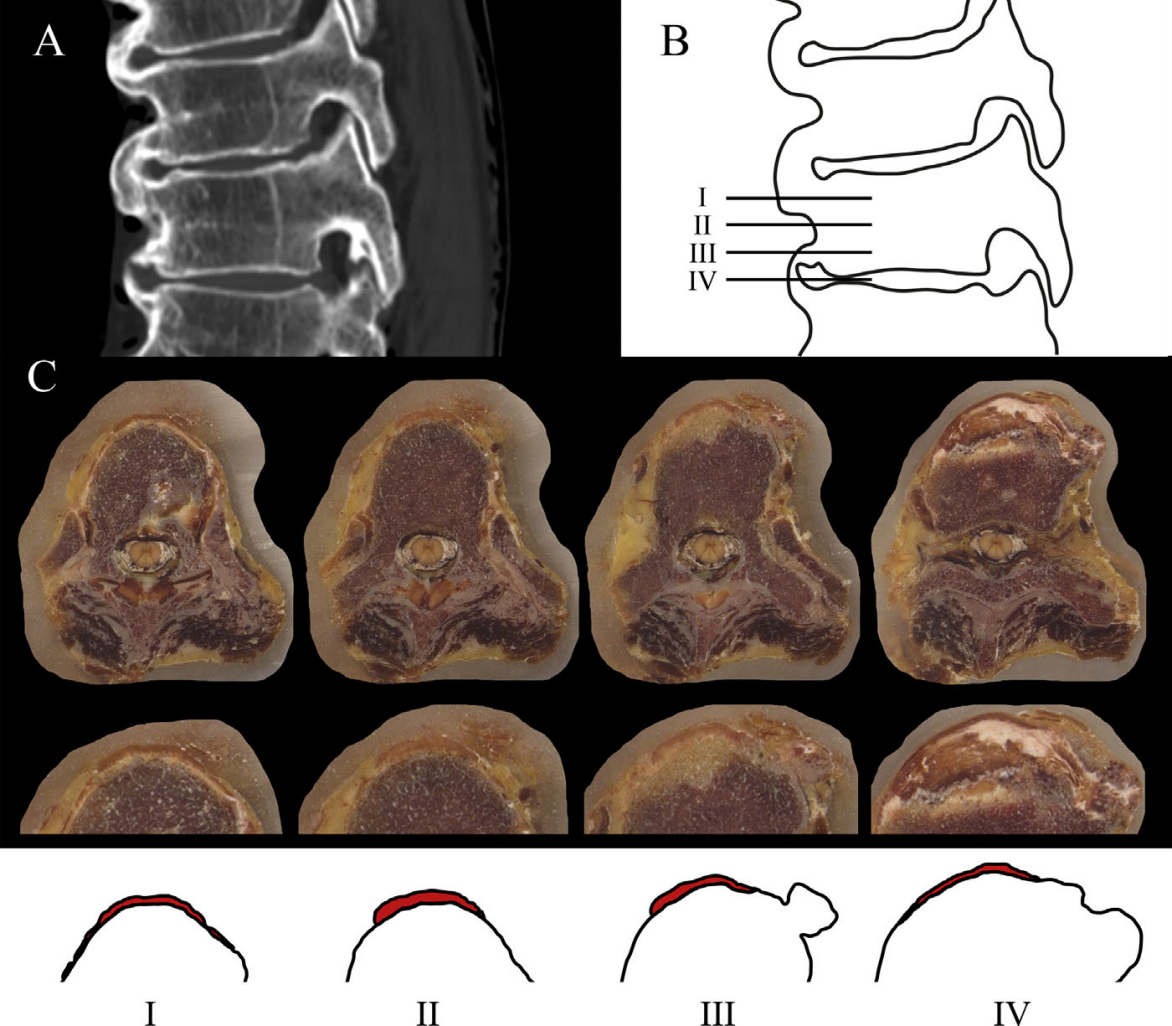
Example of the selected levels for segmentation of the ALL. In (A), the sagittal CT scan of vertebral bodies T9 to T11 is shown of a 93‐year‐old woman with bridging. In illustration (B), the horizontal lines represent the levels that were selected for segmentation of the ALL: at the level of the vertebral body adjacent to the cranial endplate (I), at the mid‐vertebral level (II) and adjacent to the caudal endplate (III) and at the level of the intervertebral disc (IV). In (C), the cryomacrotome images are shown at four levels of T8 and the T8‐9 intervertebral disc from a 92‐year‐old woman with magnification and illustration of the location of the ALL (in red). The new bone starts to appear at the level near the caudal endplate and is clearly present at the level of the intervertebral disc. CT, computed tomography; ALL, anterior longitudinal ligament.

**Figure 3:**
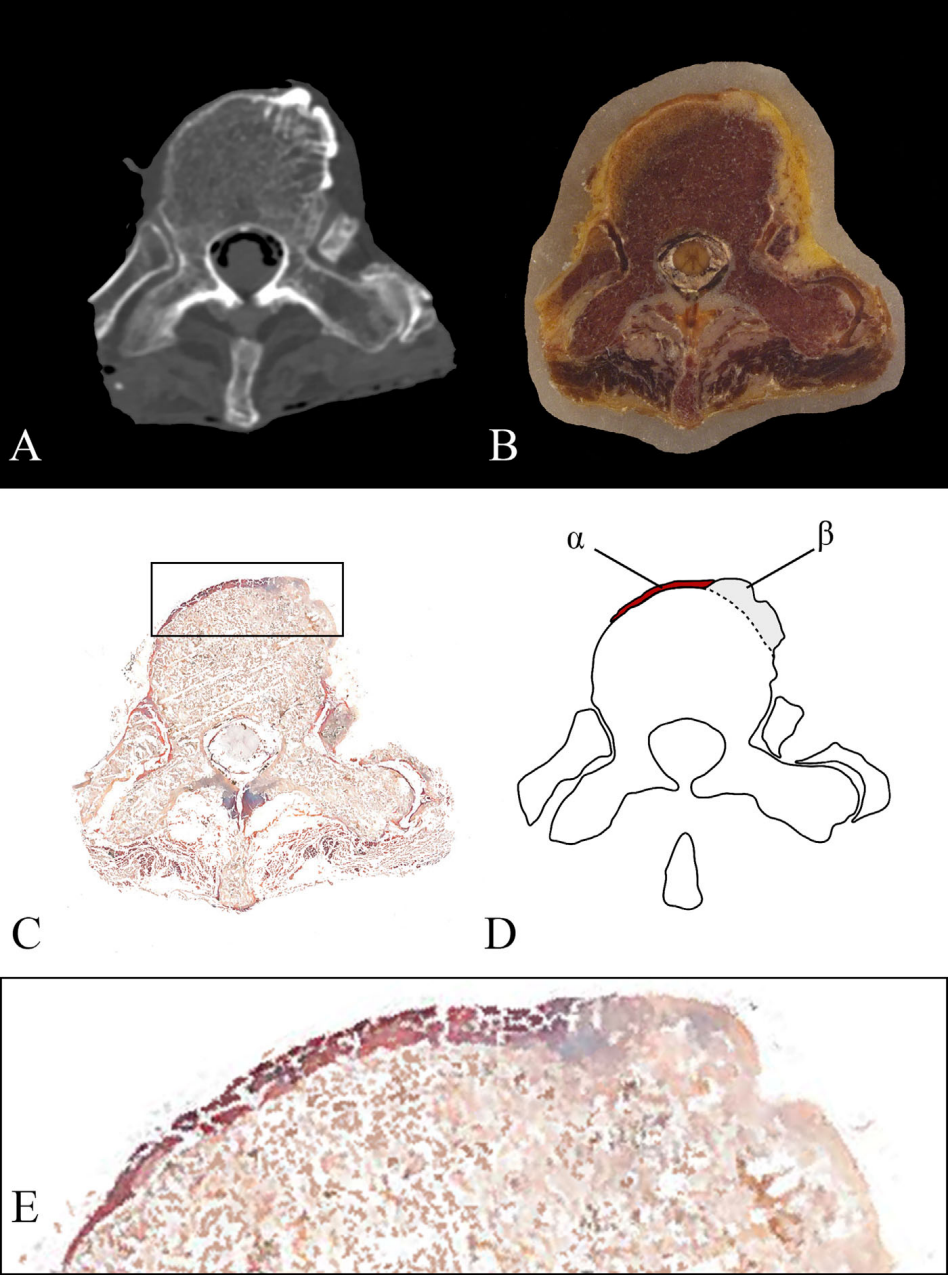
Segmentation of the anterior longitudinal ligament. The computed tomography axial view of vertebral level T10 of a 93‐year‐old woman is shown (A), with the corresponding cryomacrotome photograph (B) and section slice (C, Mallory Cason staining). Magnification of the ALL and the newly formed bone in C (box) is presented in (E). The ALL was identified based on the morphology and anatomical course using the cryomacrotome images and the slice sections after comparison with the fragments of the ALL from a specimen without DISH processed identically to the other four cadaveric spines. (D) Shows a graphic illustration with the ALL in red (α) and the new bone in gray (β). CT, computed tomography; ALL, anterior longitudinal ligament.

All photographs of the cryomacrotome sectioning were stacked in three‐dimensional fashion and registered, after coarse manual alignment in MeVisLab (MeVis Medical Solutions AG, Bremen, Germany^10^), using elastix^11^ optimizing for a rigid‐body transformation. The segmentation of the ALL was performed manually by a single observer at three levels per vertebral body and at the level of the intervertebral disc on the cryomacrotome images (Figs. 2 and 3, Appendix S1). In case the ALL was difficult to recognize in the spinal columns with DISH, the photographs could be visually compared to photographs of the ALL from the spine without DISH. Furthermore, the slice sections were stained using Mallory–Cason trichrome to discriminate between ligament and bone (Appendix S1).^12^

The segmentations of the ALL were then projected on the three‐dimensional reconstructed CT scans for each corresponding spinal column to examine its location. An orthopedic surgeon, anatomist, radiologist, and scientist, all with expertise in DISH, reviewed the location of the ALL in subjects with DISH and discussed the likelihood that the newly formed DISH ossifications originated in whole or in part from the ALL.

## RESULTS

The spines were harvested from two males (age at death 79 and 88 years) and two females (age at death 92 and 93 years) with DISH and one control male (age at death 81 years) without DISH. In the cadaveric specimens with DISH, between four and eleven complete bone bridges were present (Supplement Table S1).

### Macroscopic Anatomy

In all four DISH cases, the ALL and the newly formed bone were clearly recognizable as separate anatomical structures (Fig. 1). The ALL was estimated to be displaced about 5–15 mm from the midline, to the contralateral side of the newly formed bone, without any interruption of the longitudinal course of the ligament fibers. In the control spine, the ALL was located symmetrically in the middle of the ventral spine.

### Processed Images

**Figure 4:**
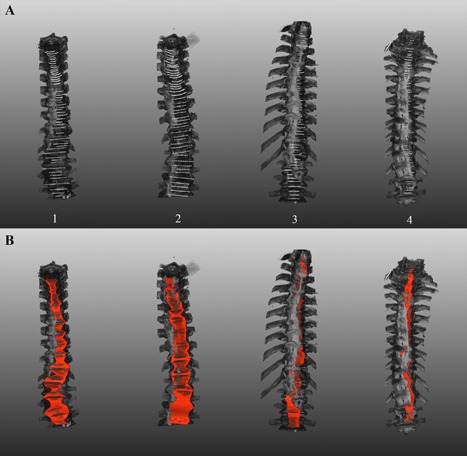
The anterior longitudinal ligament projected on the three‐dimensional reconstructed CT scans. The primary segmentation lines circumscribing the ALL are shown in white in (A). In (B), the left and right extremes of the segmentation were connected by a red color to illustrate the circumferential extend of the ALL. In (B), the curve of the ALL overlaying the vertebral body/intervertebral disc is not included in the illustration. As a result, the ALL may appear to be located within the bone; however, this is not the case. Videos of the four spines can be viewed (supplemental videos S1–S4). CT, computed tomography; ALL, anterior longitudinal ligament.

Segmentation of the ALL was achieved at all selected axial levels of the spinal columns with DISH (Fig. 4, Supplemental videos S1–S4). Where no new bone had formed the ALL was observed at its native place, mid‐anterior of the spine with symmetrical presence on the left/right side. The ALL was manually segmented in 208 axial images in total (median distance between left and right extremes 11.3 mm, range 1.8–39.2 mm) and 146 images contained newly formed bone in the four spines. The ALL was partially overgrown by new bone in 54 images (median distance 1.2 mm [16.4% of the ALL], range 0.2–3.4 mm). The new bone was formed mainly on the right side and the ALL was located primarily at the left side of the spine at the ankylosed levels. We concluded that new bone and ALL were simultaneously present in all four cadaveric spines with DISH. The possibility that the newly formed bone originated from the ALL was regarded highly unlikely as a morphologically normal ALL was present and continuous, albeit displaced by the newly formed bone in the DISH segments.

## DISCUSSION

In this study, CT imaging and cryomacrotome processing were used in cadaveric spines with DISH to explore the spatial relation between the new bone and the ALL. We concluded that the ALL was morphologically normal, and that it was displaced away from (or more likely, by) the new bone. The present findings contradict the hypothesis that DISH originates as a progressive ossification of the ALL.

The following observations supported our conclusion: (1) the ALL appeared displaced to the contralateral side in toto; (2) new bone was formed overlaying the ALL instead of being incorporated within the ALL; (3) the formation of bone did not appear to start in the centre of the ALL; (4) the new bone was located anterolaterally of the spine, not following the median course of the ALL; and (5) the ALL is generally thinner at the level of the intervertebral disc, however, in DISH new bone is most abundant at this level. Results from the current study support the theory describing ALL displacement as a result of DISH, accompanied by a potential degradation of the ALL at the location where the new bridging bone has taken over (some of) the stabilizing function of the ALL.^13^

The observation that the newly formed bone in DISH appeared to “push” the ALL to the left was described in 1950 by Forestier and in 1974 by Vernon‐Roberts.^14^, ^15^ The presumption that the formation of new bone could begin as an ossification of the ALL was proposed by Resnick et al. in 1978.^16^ They described “calcified collections of metaplastic cartilage (endochondral bone formation) within the ligament” as Type I changes. Type II changes were described as being associated with intervertebral disc alterations with extrusion of the disc in the anterior direction and subsequent progressive traction‐induced ossification adjacent to the site where the ALL is attached to the vertebral body. Additional research to explore and validate the proposed two types of changes was, however, not performed. In recent literature it has become customary to describe DISH as an ossification or calcification of spinal longitudinal ligaments, especially the ALL (17/44 DISH articles published in 2016).^5^, ^8^, ^9^ However, none of these 17 articles cited a study testing the validity of this assumption.

In all four spines, segments with incomplete bridging were also observed probably representing earlier, immature phases of DISH. At these segments, the new bone merged seamlessly with the vertebral body, suggesting the new bone to originate from the vertebral body (Fig. 3). However, more detailed research on the early developmental phase of the new bone growth, with special attention to histology, is essential to confirm this observation.

Except for the ALL of the control specimen, the cryomacrotome sectioning of a complete cadaveric spine without DISH was not included in the current exploratory study. Therefore, an objective comparison of the circumferential extent and possible degradation of the ALL in spines with and without DISH could not be performed. This study remains a purely descriptive cross‐sectional study and was limited to four cases after consideration of the labor‐intensive sample processing and highly consistent results of the first four spines.

This exploratory study described the presence, both macroscopically and through CT and cryomacrotome image analysis, of a displaced but otherwise normal appearing ALL in spinal columns with unequivocal DISH. The results imply that, contrary to current thinking, DISH may not originate from within the ALL.

## AUTHORS' CONTRIBUTIONS

Substantial contributions to research design, or the acquisition, analysis, or interpretation of data: JSK, EJMS, BP, RWH, MS, PRS, CFB, RLAWB, HHW, FCO, PAJ, JJV. Drafting the paper or revising it critically: JSK, EJMS, CFB, PAJ, JJV. Approval of the submitted and final versions: JSK, EJMS, BP, RWH, MS, PRS, CFB, RLAWB, HHW, FCO, PAJ, JJV.

## Supporting information

Additional supporting information may be found in the online version of this article.

Supporting Appendix S1.

Supporting Table S1.

Supporting Video S1.

Supporting Video S2.

Supporting Video S3.

Supporting Video S4.
