# Supplementary material for: Anterior longitudinal ligament in diffuse idiopathic skeletal hyperostosis: Ossified or displaced?
Source: J Orthop Res. 2018 May 24;36(9):2491–6. doi: 10.1002/jor.24020 (PMC6175084; doi:10.1002/jor.24020)
Supplement: Supplementary file 1 — Supporting Appendix S1. [file JOR-36-2491-s001.docx]

Supplemental data

Appendix 1

Detailed information on the computed tomography settings and sample processing

*Computed tomography settings*

All images were acquired in spiral mode using the following parameters: collimation of 64x0.625, pitch of 0.578, gantry rotation time 0.4 s, tube voltage 120 kVp, tube current 250 mAs. Corresponding volumetric CT dose index (CTDIvol) was 21.4. Images were reconstructed with a slice thickness of 0.9 and increment of 0.7 mm using standard filter C and iDose level 6 (IQon Spectral CT, Philips Healthcare, Best, The Netherlands).

*Cryomacrotome procedure*

The thoracic spines with DISH were cut into three parts (a cranial, middle and caudal section including 3 to 7 vertebral levels) to facilitate fitment of the spine in the cryomacrotome (Leica CM3600 XP, Nussloch, Germany). The three sections of each spine were placed upright in a rectangular container (300x15x18 mm) and frozen in 1% carboxymethylcellulose at minus 25 degrees Celcius. After overnight freezing, the container was removed and the frozen embedded spine was mounted in the cryomacrotome. Axial sectioning was performed automatically with 25μm slice thickness. Every five sections (125μm) the cryomacrotome was paused and a photograph was taken of the embedded sample (Leica DFC450 C, Wetzlar, Germany). Every twenty sections (500μm) adhesive tape (3M Glass Cloth Tape 365) was affixed to the frozen sample and a section of 25μm was cut that remained attached to the tape. The tapes with sectioned material were dried and in case the manual segmentation of the ALL on the cryomacrotome photographs was uncertain the tapes were stained using Mallory-Cason trichrome to confirm the location of the ALL by creating contrast between soft tissue, including the ALL, and bone [as described in ‘van Leeuwen MB, Deddens AJ, Gerrits PO, Hillen B. A modified mallory-cason staining procedure for large cryosections. *Stain Technol*. 1990;65(1):37-42.’].

Three 20x10mm rectangular fragments of the ALL of the spine without DISH were resected at three thoracic vertebral levels to compare the ALL of the spines with DISH to the ALL of a control spine. The three ALL fragments were sectioned, photographed and collected on tape in the axial, sagittal and coronal direction with equivalent settings to the spines with DISH.

*Segmentation*

The segmentation of the ALL was performed at three levels per vertebral body and at the level of the intervertebral disc (Figure 2). Steps of 25% of the total vertebral body height (below the cranial endplate, mid vertebral and above the caudal endplate) were chosen to create a general overview of the location of the ALL overlaying the vertebral body. Three observers (two researchers and an anatomist) assessed the location of the ALL in the first spine and unanimously agreed on the location and course of the ALL. For this reason, a single observer segmented the ALL in the three other spines.
