# Supplementary material for: Anterior longitudinal ligament in diffuse idiopathic skeletal hyperostosis: Ossified or displaced?
Source: J Orthop Res. 2018 May 24;36(9):2491–6. doi: 10.1002/jor.24020 (PMC6175084; doi:10.1002/jor.24020)
Supplement: Supplementary file 2 — Supporting Table S1. [file JOR-36-2491-s002.docx]

Supplement Table 1: Characteristics of the four cadaveric spines with DISH

| **Cadaver** | **Sex** | **Age at death** (in years) | **Spinal levels** | **Segments with complete bridges** | |
| --- | --- | --- | --- | --- | --- |
|  |  |  |  | Total number | Location |
| 1 | Female | 93 | T2 – L3 | 4 | T7 – T11 |
| 2 | Female | 92 | T4 – L3 | 8 | T4 – T7, T8 – T11, L1 – L3 |
| 3 | Male | 88 | T1 – L3 | 11 | T2 – L1 |
| 4 | Male | 79 | C6 – L2 | 10 | T3 – L1 |
|  |  |  |  |  |  |
